# The hermit crab's nose—antennal transcriptomics

**DOI:** 10.3389/fnins.2013.00266

**Published:** 2014-01-21

**Authors:** Katrin C. Groh, Heiko Vogel, Marcus C. Stensmyr, Ewald Grosse-Wilde, Bill S. Hansson

**Affiliations:** ^1^Department of Evolutionary Neuroethology, Max Planck Institute for Chemical EcologyJena, Germany; ^2^Department of Entomology, Max Planck Institute for Chemical EcologyJena, Germany

**Keywords:** crustacea, antennules, olfaction, molecular evolution, genomics

## Abstract

In the course of evolution, crustaceans adapted to a large variety of habitats. Probably the most extreme habitat shift was the transition from water to land, which occurred independently in at least five crustacean lineages. This substantial change in life style required adaptations in sensory organs, as the medium conveying stimuli changed in both chemical and physical properties. One important sensory organ in crustaceans is the first pair of antennae, housing their sense of smell. Previous studies on the crustacean transition from water to land focused on morphological, behavioral, and physiological aspects but did not analyze gene expression. Our goal was to scrutinize the molecular makeup of the crustacean antennulae, comparing the terrestrial *Coenobita clypeatus* and the marine *Pagurus bernhardus*. We sequenced and analyzed the antennal transcriptomes of two hermit crab species. Comparison to previously published datasets of similar tissues revealed a comparable quality and GO annotation confirmed a highly similar set of expressed genes in both datasets. The chemosensory gene repertoire of both species displayed a similar set of ionotropic receptors (IRs), most of them belonging to the divergent IR subtype. No binding proteins, gustatory receptors (GRs) or insect-like olfactory receptors (ORs) were present. Additionally to their olfactory function, the antennules were equipped with a variety of pathogen defense mechanisms, producing relevant substances on site. The overall similarity of both transcriptomes is high and does not indicate a general shift in genetic makeup connected to the change in habitat. IRs seem to perform the task of olfactory detection in both hermit crab species studied.

## Introduction

Crustaceans successfully conquered a variety of habitats, including backwater and freshwater as well as marine and terrestrial habitats. At least five lineages of crustaceans independently succeeded in the transition from water to land (Bliss and Mantel, 1968; Powers and Bliss, 1983). Such substantial changes in environment require extensive adaptation, for example regarding metabolism, water and ion balance, and behavior. Another obviously affected area are the acoustic-, visual and chemical senses, due to the differences in chemical and physical properties of the medium conveying the respective stimuli. In crustaceans antennules and antennae are important organs for sensory tasks; both can for example detect mechanical stimuli. Additionally, the antennules are the main chemosensory organ (Bush and Laverack, 1982; Cate and Derby, 2002). In terms of chemosensation, lobsters are the primary decapod models. Lobster antennulae are the olfactory organs, while the sense of taste is located on their walking legs (Atema, 1977; Derby and Atema, 1982; Johnson et al., 1988). The most numerous sensillum type on the antennules are aesthetascs, unimodal chemosensory sensilla located on the last antennular segment (Ghiradella et al., 1968a). Aesthetascs each house approximately 300 olfactory sensory neurons (OSNs) and are the place of odor detection (Gleeson, 1982). In the marine hermit crab *Pagurus bernhardus* the aesthetascs are long and slender (Hansson et al., 2011) while in its terrestrial relative *Coenobita clypeatus* they are short and blunt (Krång et al., 2012). This change is probably an adaptation to function in air rather than water, comparable to its terrestrial relatives *Coenobita compressus* (Ghiradella et al., 1968a,b) and *Birgus latro* (Stensmyr et al., 2005). The OSNs from all aesthetascs converge into one antennal nerve leading to the olfactory lobe (OL), the primary central processing center of olfactory input. The OL is composed of column-like glomeruli, approximately 560 in *P. bernhardus* and 1000 in *C. clypeatus* (Koczan, 2012). In the latter the OL is enlarged, consists of two sublobes and dominates the brain in size, while the volume of visual or mechanosensory processing centers is similar to their aquatic relatives (Harzsch and Hansson, 2008). Investigations of the terrestrial hermit crab *Birgus latro*, a close relative of *C. clypeatus*, suggested that the adaptation of the olfactory organs is insect-like (Rittschof and Sutherland, 1986; Greenaway, 2003; Stensmyr et al., 2005).

Contrary to insects, information on the molecular biology of the crustacean olfactory system is sparse. In contrast, the insect chemosensory system is very well-described. Here, relevant genes include two classes of soluble proteins found in the sensillum lymph; odorant binding proteins (OBPs) and chemosensory proteins (CSPs); while CSPs have been reported in all groups of Arthropoda, OBPs are limited to Hexapoda (Vieira and Rozas, 2011). As regards the relevant receptors, one chemoreceptor superfamily containing olfactory receptors (ORs) and gustatory receptors (GRs), and a receptor class named ionotropic receptors (IRs) are known [for reviews see Vieira and Rozas (2011), Silbering et al. (2011)]. GRs which also have been identified in all major groups of Arthropoda are considered to be ancestral to the Hexapoda specific ORs (Robertson et al., 2003). GRs are expressed in insect antennae but also in other head and body appendages (Montell, 2009); GR-expressing neurons of insects respond to tastes like sugar or bitter compounds, but also to CO_2_ (Kwon et al., 2006; Robertson and Kent, 2009). IRs are currently considered the most ancient chemoreceptors. They derived from the ionotropic glutamate receptors early in the protostomian lineage and are comprised of two groups based on expression data (Benton et al., 2009). While the antennal IRs of insects are involved in the detection of e.g., acids, amines, aldehydes, and alcohols, the divergent IRs are not expressed in insect olfactory organs and their function is so far unknown (Benton et al., 2009; Ai et al., 2010; Croset et al., 2010; Silbering et al., 2011). The most ancient IRs known to date are IR25a and IR93a, both belonging to the antennal IR subgroup. Insect odor specific IRs are always expressed together with IR25a or its homolog IR8a, which functions as a coreceptor in IR-expressing dendrites of *Drosophila melanogaster* (Croset et al., 2010).

*Daphnia pulex* is the only crustacean with near-complete genome data available. The data contains CSPs, GRs, and IRs, with most of the latter belonging to the divergent IR subgroup (Penalva-Arana et al., 2009; Croset et al., 2010; Vieira and Rozas, 2011; Kulmuni and Havukainen, 2013). Further indication of IR based olfaction in crustaceans was obtained from lobsters, identifying the homologs of IR25a, IR93a, and IR8a and showing their expression in OSN clusters (Hollins et al., 2003; Corey et al., 2013).

As mentioned, the transition to land has consequences for other functions beyond the chemical sense. The exposed position of crustacean antennae necessitates protection from injury and pathogens. While the cuticle, mechanosensors and withdrawal reflex account for the first, the second has not been studied in this tissue yet. Nevertheless, besides fruit flies, the immunosystem of decapod crustaceans is the most intensely studied among arthropods. The first step of pathogen defense is non-self recognition; in invertebrates mediated for example through pattern recognition proteins such as C-type lectins. Lectins recognize and bind sugars to agglutinate cells which are in turn recognized and destroyed by the innate immune system, involving the prophenoloxidase (proPO) activating system and apoptosis (Soederhall and Cerenius, 1998; Cerenius et al., 2010). Another important factor of the arthropod immune system are antimicrobial peptides (AMPs). The expression of insect AMPs is in some cases up-regulated upon microbial challenge and subsequently negatively affects microbial growth. In crustaceans many kinds of AMPs have been described, including both constitutively expressed and inducible ones (Han-Ching Wang et al., 2010). There is indication that their regulation involves the Toll or the imd pathway, similar to the situation found in insects (Cerenius et al., 2010; Han-Ching Wang et al., 2010). Possible components of both pathways have been identified in shrimp species, including *Toll, imd, spätzle, relish, and dorsal* (Cerenius et al., 2010), but details about the extent and specificity of AMP regulation by either pathway remain to be investigated.

Previous studies on the crustacean transition from water to land considered morphological, behavioral and physiological aspects, but did not include analysis of gene expression (Bliss and Mantel, 1968; Powers and Bliss, 1983; Greenaway, 2003). Our goal was to scrutinize molecular adaptation in the antennae of terrestrial hermit crabs. Therefore, we selected adults of the hermit crab species *Coenobita clypeatus* (Herbst 1791; Paguroidea, Coenobitidae) as a representative of the genus Coenobitidae, which has planktonic larvae and a fully terrestrial adulthood for comparison of their molecular makeup with adults of the marine species *Pagurus bernhardus* (L. 1758, Paguroidea, Paguridae). According to recent investigations based on a combined analysis of molecular, morphological, and fossile data, the ancestral lineages of both species split approximately 173 Mya ago (Bracken-Grissom et al., 2013). We generated antennal transcriptome data using 454 and Solexa/Illumina technology. The data was assembled and investigated from the abstract level of GO annotation. Furthermore, we employed BLAST searches based on homology comparison and HMM profile prediction to screen selectively for genes involved in chemo- and mechanosensing, neuronal signaling, and immune response, and interpret the results in the context of known and prospective abilities of crustaceans.

## Materials and methods

### Animal collection and dissection

#### Pagurus bernhardus, coenobita clypeatus

Specimens of *P. bernhardus* were procured from the Biologische Anstalt Helgoland (Germany) and *C. clypeatus* specimens were ordered from Peter Hoch Import—Export Waldkirch (Germany). Both examined species are neither endangered nor protected. Adult animals of both sexes were dissected at the SLU Alnarp (Sweden); specimens were cold anesthetized and antennulae cut off using scissors pooling the antennules of each species. Dissected antennulae were shipped to Jena (Germany) on dry ice for RNA extraction.

### Sequencing, read cleanup, and de novo assembly

For RNA extraction tissues were cooled further over liquid nitrogen. The frozen tissue was transferred to a liquid nitrogen cooled mortar and ground. The homogenate was covered with 1 ml TRI reagent (Sigma-Aldrich, St. Louis, MIS). Further steps were performed according to the manufacturer's instructions, replacing choloroform with 1-bromo-3-chloro-propane as recommended by Sigma-Aldrich to lower toxicity and for better phase-separation. For ethical reasons we restricted the number of specimens according to the minimal amount of RNA required. Including both sexes this meant 10 pairs of antennules for *C. clypeatus* and 5 pairs for *P. bernhardus*. Two micrograms of total RNA preparations were sent to Evrogen, Moscow (Russia) for production of normalized cDNA. The normalized cDNA was afterwards sequenced by AGOWA, Berlin (Germany) by 454 sequencing on an GS FLX+ sequencer, for 400 bp single read length. An additional RNA sample was not normalized, reverse transcribed and sequenced by Solexa/Illumina for 76 bp single end reads on an Illumina Genome Analyzer II. Bustard 1.3.2 was used for basecalling and Firecrest was employed for offline image analysis. Raw sequencing data is available for download (EBI/ENA Study acc. number ERP002374, http://www.ebi.ac.uk/ena/data/view/ERP002374). Data was screened for contaminants and linker/adapter sequences, followed by *de novo* hybrid assembly of the sanitized reads in CLC genomics workbench V 5.5. Reads from both sequencing techniques were used simultaneously without removal of duplicates. Thorough contaminant cleanup was made necessary because of high contamination of raw 454 data by reads originating from various environmental taxa. Bioinformatic statistics were performed using scripts provided at http://manuals.bioinformatics.ucr.edu by Thomas Girke updated 28th of January 2010 and a script by Joseph Fass revised 2010 (c) 2009 The Regents of University of California, Davis Campus.

#### Manduca sexta

As a reference for arthropod antennal transcriptome data we chose the 454 sequenced antennal transcriptome of *M. sexta* published by Grosse-Wilde (Grosse-Wilde et al., 2011). We performed a new assembly of the data using CLC 5.5. For better comparativeness we used the same parameters as for the hermit crab datasets.

### Annotation

We created BLAST2GO databases based on each dataset and performed homology searches after dynamic translation (BLASTX) against non-redundant databases (National Center for Biotechnology Information, NCBI), using the default cutoff parameters of BLAST expectation value (1.0E-3), InterProScan and assigned KEGG maps. Annotation was based on an *E*-Value—Hit-Filter of 1.0E-6. Additionally we carried out BLAST searches based on HMM profile prediction (*E*-value cutoff 1.0E-3) and homology comparison (*E*-value cutoff 1.0E-1) of selected reference gene sequences against the datasets. In the following, “EST” will refer to annotated contigs.

### Bioinformatics

To compare the transcriptomes, B2GO combined graphs were drawn for all datasets and in all the three categories: cellular component, molecular function, and biological process. The cutoff of sequence count was set according to the maximum of possible nodes. If a term turned up in at least one species but did not in one or both of the others, annotation was checked manually and the number of sequences was added as necessary. We calculated the percentage of ESTs assigned to one term to the total count of ESTs assigned to terms on annotation level 2 or 3, respectively, by the following formula: (number of ESTs assigned to the term of interest * 100)/(number of ESTs assigned to terms on level x). For further comparison we used R 2.15.2 (http://cran.r-project.org/bin/windows/base/old/2.15.2/) to plot the respective charts, test, and compare the categories in the species and the ratios or presence and absence of terms. Dendrograms were compiled using the MUSCLE alignment tool (Edgar, 2004) followed by FastTree2 dendrogram calculation (Price et al., 2010). Adobe Illustrator CS5 was employed to compile the figures.

### RACE PCR

Selected candidate gene fragments were extended using SMARTer and Marathon RACE-PCR kits (Clontech) following the respective manufacturer's instructions and specifications. Gene specific primers were designed with the online input version of primer3 (http://frodo.wi.mit.edu/).

## Results

### Sequencing and assembly

For transcriptome data generation, antennal DNA samples from *Coenobita clypeatus* and *Pagurus bernhardus* were sequenced using Roche 454 and Solexa/Illumina technologies. Reads were sanitized and 454 data was additionally cleaned from reads associated to contaminants like algae, fungi, and bacteria, the cutoff being set at *E* < 10–20 (Table 1).

**Table 1:** **Number of contaminating sequences detected in and removed from 454 sequencing reads of both species; separated by taxids (ncbi)**.

**Taxid**	**Taxon name**	***P. bernhardus***	***C. clypeatus***
2	Bacteria	1316	357
2157	Archaea	231	10
2736	*Verrucomicrobium spinosum*	2057	1234
3041	Chlorophyta	2469	2241
4751	Fungi	15,108	1592
33,634	Stramenopiles	294	458
38,254	Glaucocystophyceae	10,637	2742
554,915	Amoebozoa	1126	536
Remaining reads		270,565	432,240

**Figure 1:**
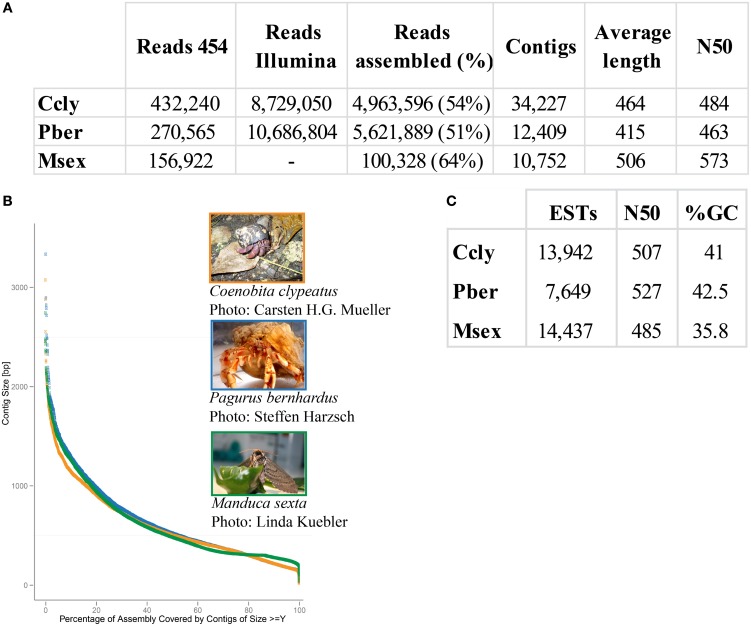
**Summary statistics sequence assembly. (A)** Read numbers, contig numbers, and average values of CLC assembly **(B)** contig length distribution, orange: *C. clypeatus*, blue: *P. bernhardus*, green: *M. sexta* **(C)** assembly statistics final datasets, *P. bernhardus*/*C. clypeatus*: contigs with an average coverage above 5, *M. sexta*: contigs above 200 and single reads above 300 nucleotides.

Approximately 30% of the raw 454 sequencing data of *P. bernhardus* was removed, leaving 270,565 reads to proceed. The raw 454 sequencing data from *C. clypeatus* additionally contained internal linker sequences and was therefore split and sanitized. Five reads were split into 4 parts, 567 into 3 parts and 31,766 into 2 parts. After sanitization and contaminant cleanup 432,240 single reads remained. Assembly resulted in 34,227 contigs with an average length of 464 nucleotides for *C. clypeatus* and 9788 contigs with an average length of 415 for *P. bernhardus* (Figure 1). A higher total contig count in *C. clypeatus* was assembled from a smaller number of sequencing reads than in *P. bernhardus* (Figure 1A). Nevertheless, the N50 value is similar and the distribution of assembly coverage shown in Figure 1B is almost identical. The N50 values as well as the contig distribution in both hermit crab species are highly similar to *M. sexta* transcriptome (Figures 1B,C). Summary assembly statistics of the two datasets confirmed a quality similar to previously published datasets on insect olfactory tissues, allowing comparison. To eliminate problems caused by underrepresentation of critical sequences, contigs with an average coverage of less than 5 were ignored in the BLAST2GO annotation but included for survey of specific gene families, performing manual curation. The datasets for the GO comparison of both transcriptomes finally yielded 13,942 unique contigs for *C. clypeatus* and 7649 contigs for *P. bernhardus*, respectively (Figure 1C). For quality assessment we included a reassembly of a previously published antennal transcriptome dataset from the insect *Manduca sexta* containing contigs and single reads above a cutoff of 300 nucleotides.

### GO annotation

GO classification provides a general and transferable vocabulary for abstract description of functions and processes present within a given dataset (Ashburner et al., 2000). We performed GO annotation of the transcriptome data using BLAST2GO as described in Conesa et al. (2005). Again we included *M. sexta* data for comparison, performing a reanalysis. A total of 3453 contigs (25%) in the *C. clypeatus* dataset and 2505 contigs (33%) in *P. bernhardus* yielded a suitable BLAST result. Domain identification using InterProScan (IPS) yielded matches for 8369 contigs (60%) of *C. clypeatus* and 4717 contigs (62%) of *P. bernhardus*. Taken together this indicates a large number of sequences with no clear ortholog, but identifiable functional domains. For comparison, reanalysis of *M. sexta* yielded 8771 contigs with BLAST hit (61%) and 7943 contigs (55%) with IPS matches.

For distinction purposes we refer to the dataset generated from the *Pagurus bernhardus* transcriptome as “Pber,” to the *Coenobita clypeatus* dataset as “Ccly” and to the *Manduca sexta* dataset as “Msex.”

**Figure 2:**
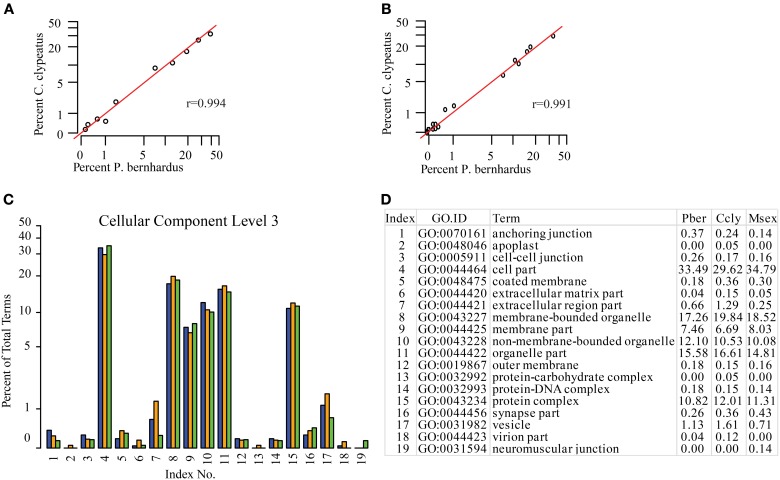
**Distribution of GO terms in the category Cellular Component. (A)** Cellular Component level 2, **(B)** Cellular Component level 3. Red line: predicted linear slope. *r* = Pearson coefficient, **(C)** Barplot Cellular Component level 3, all terms; blue: Pber, orange: Ccly, green: Msex; **(D)** chart index table.

Since the function of the antennal tissues is probably very similar but not identical in all three species, we assigned KEGG maps to compare the representation of their main molecular functions. As expected they are equally well-represented in the three datasets (data not shown). Based on the GO terms assigned to contigs we compared the species regarding different categories and on different annotation levels (full list in Supplementary Table S1). One potential problem of GO annotation affecting comparisons is an over- or underrepresentation of ESTs resulting in a shift of the EST ratio assigned to specific terms on a distinct level. To address this issue we compared the representation of terms between the datasets and calculated the Pearson coefficient as a measure of similarity. If annotation of the two compared datasets is not adversely affected by this issue the correlation coefficient should be close to 1 for the category of “Cellular Component,” since it is reasonable to assume similar term distribution here. Comparing Ccly and Pber the coefficient is 0.994 on level 2 and 0.991 on level 3, respectively (Figures 2A,B), confirming comparability. Therefore, we assume that there is no overall shift in the category representation caused by over- or underrepresentation of terms. In contrast to “Cellular Component,” “Biological Process,” and “Molecular Function” could be affected severely by the differences in lifestyle. However, correlation coefficients for the same analysis of “Biological Process” and “Molecular Function” on level 2 indicate no difference while on level 3 there is a Pearson coefficient of 0.989 for “Biological Process” and 0.970 for “Molecular Function,” indicating slight different abundance and a potential shift in function. Based on the distribution of GO terms and their proportions we designed comparative graphs depicting the comparison of single terms with respect to their percentages. At the compelling level 3 of “Cellular Component” we find slight differences for example in the highest abundant term “cell part” (33.5 and 29.6%) (Figures 2C, D). While Ccly has a higher portion of “membrane bounded organelle” (19.8%) compared to Pber (17.3%), “non-membrane-bounded organelle” associated sequences represent a larger part of the Pber dataset (12.1%) than it does in Ccly (10.5%).

To gain deeper insights into the species-specificity of terms we compared the number of independent occurrences of terms in the categories “Biological Process” and “Molecular Function” on level 3. Both categories contained terms exclusively associated with Msex but as we want to focus on the crustaceans we will not discuss them further.

**Figure 3:**
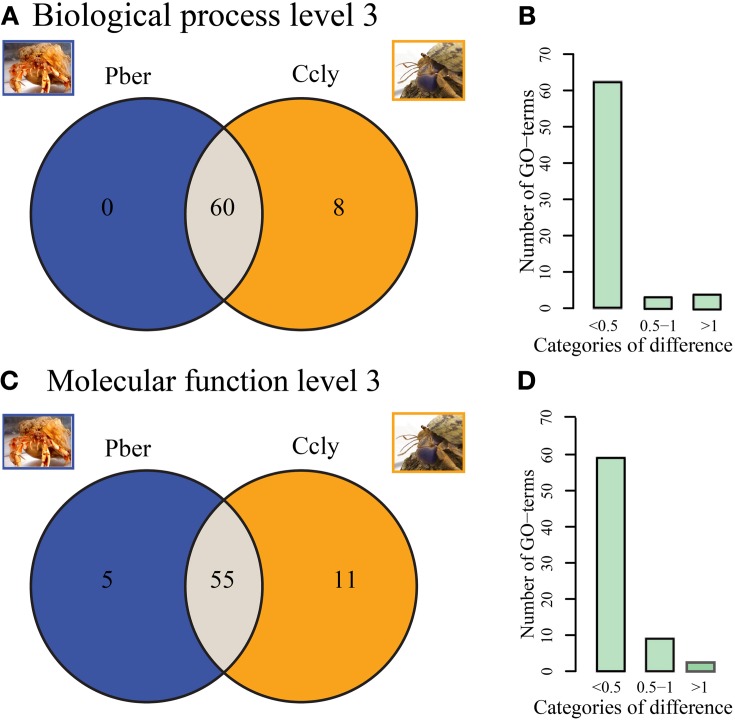
**(A,B)** Biological Process. **(A)** Distribution of assigned terms in the category of “Biological Process” between Pber and Ccly, 60 terms shared between Pber and Ccly, 8 terms unique to Ccly. **(B)** Distribution of term ratios compared between in the categories “<0.5% difference,” “0.5–1% difference,” and “>1% difference.” **(C,D)** Molecular function **(C)** Distribution of terms between Pber and Ccly, 55 terms shared, 5 terms unique to Pber, 11 terms unique to Ccly. **(D)** Distribution of term ratios in the categories “<0.5% difference,” “0.5–1% difference,” and “>1% difference.” Percentage difference has been calculated as described in Bioinformatics.

Figure 3A depicts Venn diagrams representing the counts of level 3 “Biological Process” terms which are either common to both or unique to one hermit crab species. The vast majority (88%) of terms is common to both datasets. Eight terms are limited to Ccly. While only few ESTs are assigned to most of them, the term “response to other organism” represents 0.3% of the total level 3 terms. Pber showed no unique terms on level 3 “Biological Process.” Additionally we categorized all terms observed in at least one species, relative to the proportion of the term in direct comparison between species (Figure 3B). The majority of terms differ only slightly in representation, exhibiting less than 0.5% difference. Two terms differ by 0.5–1% and 3 are in the category above 1%. A majority of terms (77.5%) is common to both datasets regarding third level terms in the category “Molecular Function” (Figure 3C). Pber shows 5 unique terms all of which have a low number of assigned ESTs. In Ccly 11 terms were unique, each with only very few ESTs assigned. The category of differences between 0.5 and 1% (Figure 3D) contained nine terms while three terms exhibited a difference bigger than 1%.

### Biological process

**Figure 4:**
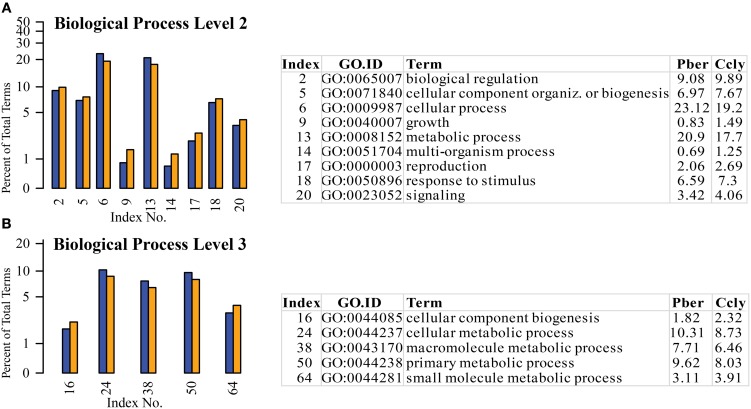
**Selected terms of the category “Biological Process” with ratio differing by at least 0.5% between the species. (A)** Level 2, **(B)** level 3; Blue bars: Pber, orange bars: Ccly. Numbering of bars indexed in the chart, proportions in the species given in per cent.

As already described in the chemosensory tissues of insects (Legeai et al., 2011), terms assigned to basic cell functions like cellular and metabolic processes had the highest representation in all three species. We also identified well-represented terms typically expected in sensory tissue like “response to stimulus” and “signaling” as initially described for *M. sexta* (Grosse-Wilde et al., 2011) also in the hermit crab datasets. The distribution of terms in most of the categories showed visible differences, so we selected the most obvious ones with a difference above 0.5% between the hermit crabs to go into further detail (Figure 4). At level 2 the highest difference in abundance was observed concerning the term “cellular process” with 23% Pber and 19% Ccly (Figure 4A). We found 18 of its child terms on level 3, where only “cellular component biogenesis” and “cellular metabolic process” differed according to the selected range of more than 0.5%. On level 2 the 2nd highest differentially expressed term was “metabolic process” with 19.2% in Pber and 17.3% in Ccly. Besides the term “cellular metabolic process” being child term of “cellular process” as well, of its 6 level 3 child terms matched by our data 2 were also differentially represented in our dataset, “macromolecule metabolic process” and “primary metabolic process” (see table in Figure 4B).

### Molecular function

**Figure 5:**
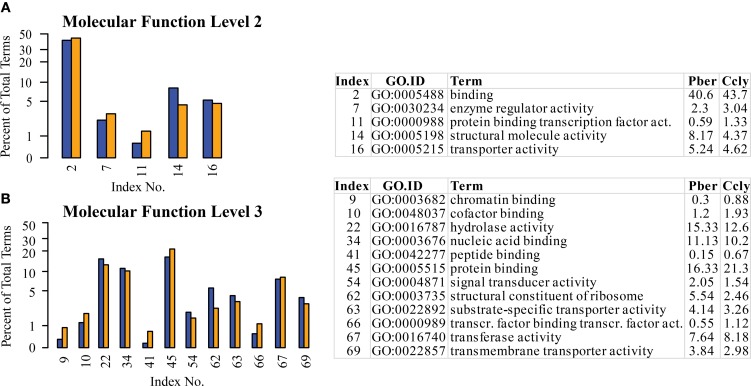
**Selected terms of the category “Molecular function” with ratio differing by at least 0.5% between the species. (A)** Level 2, **(B)** level 3; Blue bars: Pber, orange bars: Ccly. Numbering of bars indexed in the chart, proportions in the species given in per cent.

The highest representation in all three species was connected to functions in the enzymatic activity and transport terms “binding” and “catalytic activity” confirming the observations in chemosensory tissues of other species (Legeai et al., 2011; Jacquin-Joly et al., 2012). Again we selected the most obvious terms differing between species (Figure 5). At the 2nd level the most pronounced differences occurred in the categories “structural molecule activity” (8.2% Pber, 4.4% Ccly) and “binding” with 40.6 and 43.7%, respectively (Figure 5A). This was also reflected by the higher levels, where one child term of “structural molecule activity” and 4 child terms of “binding” display differing ratio between the species. The highest was observed in the term “protein binding” with 16.3% Pber and 21.3% Ccly (Figure 5B). Looking at further annotation levels only a few terms show an obviously higher EST count in Ccly compared to Pber, most differences are small and finally sum up to the observed value. Regarding level 4 child terms of “protein binding” having higher proportions in Ccly compared to Pber include “cation binding,” “hydrolase activity, acting on acid anhydrites,” “nucleotide binding” and terms connected to transferase activity.

### Selected gene families of sensing and defense

Beyond the abstract level of GO terms we focused on olfaction, the main sensory task of the antennules. One advantage of using the GO annotation in terms of comparison and transferability is the abstract level of its vocabulary. However, for a more conclusive analysis we scrutinized annotation of single ESTs, manually revising annotations where necessary. This involved homology search on nucleic acid and translated RNA level, including contigs that were not annotated by BLAST2GO.

#### Chemosensation

First we looked at the gene families of ORs, GRs, OBPs, CSPs, and SNMPs, all of which are known to be main components of chemosensory detection in insects. While we were able to mostly replicate and expand on the previously published results for *M. sexta* using our new assembly, we were not able to identify any members of these families in the crustaceans. CSPs are binding proteins implicated in chemosensing that in contrast to OBPs are present not only in insects but also in genomic data of other arthropods (Vieira and Rozas, 2011; Kulmuni and Havukainen, 2013). However, the antennal transcriptomes of both hermit crabs did not contain any contigs potentially encoding CSPs.

**Figure 6:**
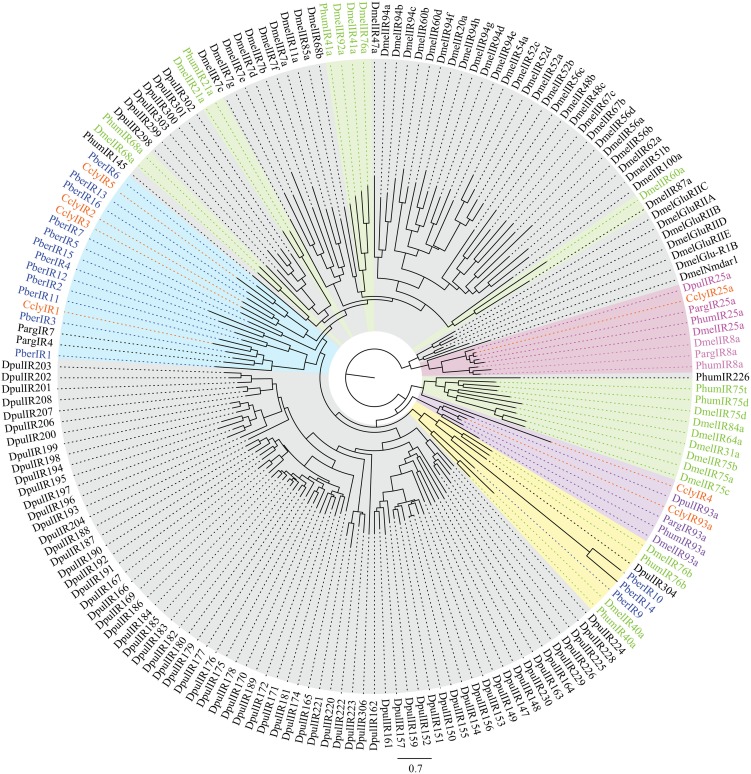
**Dendrogram of candidate IRs in *C. clypeatus* (orange) and *P. bernhardus* (blue) including reference sequences from *D. pulex* and *D. melanogaster*; highlighted in pink: ancient IRs and coreceptors, green and highlighted in green: antennal (olfactory) IRs, highlighted in blue: clusters of hermit crab candidate IRs, highlighted in yellow: cluster of both, antennal and divergent IRs containing hermit crab candidate IRs; modified after Croset et al. (2010)**.

Second we looked at IRs, ancient receptors present throughout the protostomian lineage (Croset et al., 2010). The *C. clypeatus* transcriptome yielded 20 IR candidates identified by homology comparison, including the conserved IR25a and IR93a. In *P. bernhardus* 18 IR candidates are present, also including homologs of IR25a and IR93a. Both IR25a and IR93a of *C. clypeatus* were extended by RACE PCR although not to full length. Due to RNA material limitations this was not possible for *P. bernhardus* candidates. For higher reliability only putative IR coding contigs spanning at least two of the three transmembrane domains (Benton et al., 2009) without in frame stop codons or ambiguities were selected for the calculation of a multiple sequence alignment and the dendrogram shown in Figure 6 (*C. clypeatus*: 7 candidates, *P. bernhardus*: 15 candidates). We decided to name them CclyIRX and PberIRX with X being ascending arabic numerals for discrimination where no clear homolog IR was available. Most of the hermit crab IR candidates group together with the non-coreceptor IRs from *P. argus* among the divergent IRs. PberIR9 seems to be close to the conserved antennal IR40a, a receptor with so far unknown ligand spectrum. The coreceptor IR8a was absent in both species.

#### General GPCRs

We searched for G-Protein coupled receptors (GPCR) as this ancient and highly conserved protein family is involved in a broad variety of signal transduction processes. Any influence in genetic makeup between the Pber and Cclyp should be reflected here. In the *C. clypeatus* dataset we could identify a tyramine/octopamine receptor and several contigs assigned to GPCRs, including a rhodopsine like GPCR, relaxin receptors, one neuropeptide FF receptor2 and a homolog of methuselah. The *P. bernharus* dataset yielded a relaxin receptor and a tyramine-octopamine receptor.

#### Neuronal modulation and signaling

While our methods do not allow analysis of short neuropeptides, we could scrutinize receptors and enzymes involved in neuronal processes. The *Pagurus* dataset contained ESTs annotated as dopamine beta hydroxylases, the already mentioned tyramine/octopamine receptor and a histamine gated chloride channel. In the *Coenobita* dataset several contigs were annotated as dopamine beta hydroxylases or tyrosine/dopamine beta hydroxylases; one dopamine transporter and a dopamine receptor interacting protein belonging to the heat shock protein (hsp) family could be assigned. Furthermore, a guanylyl cyclase was identified. While neither GABAa nor GABAb receptors could be found in the transcriptomes, several GABAa associated proteins and modulators were present.

#### Mechanosensation, thermosensation

Bi- or even multimodal sensilla are widely distributed among arthropods and can be found on almost all body surfaces. Among decapods, bimodal chemo-mechanosensilla are common and morphologically well-described, playing a role in close-range sensing (Cate and Derby, 2001). While the numerous cilia sensitive to mechanical stimuli on the crustacean antennules have been studied (Atema, 1977), molecular information is lacking. A recent study in *Drosophila* larvae screened for ESTs which are necessary to maintain the ability to sense touch events (Tsubouchi et al., 2012). According to BLAST searches based on these sequences the *Coenobita* dataset contained one EST with similarity to nompC, one chloride channel b and iGluRs of the NMDA2 type. In *P. bernhardus* only homologs to NMDA2 receptors could be found, confirming the presence of receptor transcripts with similar features. Thermosensing in *D. melanogaster* is mainly based on the TRP channels painless and TRPA1 (Tracey et al., 2003; Rosenzweig et al., 2005). Both are expressed in the arista, making it likely to expect similar receptors also in the crustacean antennules (Montell, 2009). However, neither homologs to painless or TRPA1 nor other TRP channels were present in our datasets.

#### Immune response and antimicrobial defense

An organ or a tissue exposed to the outside likely needs protection against potential pathogens. As the antennae have not been characterized as central production organs of immune-relevant factors we were looking broadly for expression of genes that have been considered to contribute to immune responses in crustaceans [for review see Cerenius et al. (2010)]. We were able to identify a variety of AMPs, a potent group of immune effectors [reviewed in Rosa and Barracco (2010)] and several ESTs annotated to genes which are of putative relevance in the crustacean immune system. A summary list is given in Table 2.

**Table 2:** **Potentially immune relevant ESTs, categories selected after Robalino et al. (2007)**.

	***P. bernhardus***	***C. clypeatus***
**ANTIMICROBIAL**		
C-type Lectin	10	7
Crustin	12	4
Carcinin	0	1
Anti-LPS factor	5	3
**CELL ADHESION**		
Cadherin 23	0	1
Integrin alpha	0	2
Integrin beta	1	0
Integrin beta binding protein	0	1
Peroxinectin	4	2
Tetraspanin	3	4
**CELL DEATH**		
Autophagy protein 9	0	3
**OXIDATIVE STRESS**		
Glutathione S-transferase	10	12
Peroxiredoxin	7	4
Thioredoxin	2	2
**PROTEASES**		
Aminopeptidase	1	8
Cathepsin A	0	2
Cathepsin B	2	2
Cathepsin L	1	1
Cubilin protease	1	2
**ProPO CASCADE**		
Prophenoloxidase	3	0
PO activating factor	7	6
**PROTEASE INHIBITORS**		
Serine protease inhibitor	10	17
**RNAi**		
Armitage	0	1
Tudor staphylococcal nuclease	1	3

## Discussion

Here we present the first transcriptome analysis of crustacean olfactory tissues, providing a comparison between an aquatic and a fully terrestrial species; *Pagurus bernhardus* and *Coenobita clypeatus.*

Summary assembly statistics of the two datasets confirmed a quality similar to previously published datasets on insect olfactory tissues and our reference transcriptome of *M. sexta*, allowing comparison. In agreement with previous non-olfactory transcriptome studies on crustacea, only a small number of ESTs (Ccly 25%, Pber 33%) was significantly similar to known genes in general homology searches, while InterproScan analysis assigned functional domains to ca. 60% of the ESTs in both datasets. This discrepancy most likely originates from a lack of information on crustacean molecular biology, amplified further by a lack of analysis of antennally expressed genes in general. Comparability of the crustacean datasets is further confirmed by the equality of term distribution in the GO term category of “Cellular Component.” Differences observed in the category “Biological Process” were overall small and mainly caused by a differing number of subtypes or variants of factors and regulators. However, meaningful differences were observed in the category of “Molecular Function.” For example, the 3rd level term “protein binding” is associated to nearly twice as many ESTs in *C. clypeatus* than in *P. bernhardus*. Nevertheless, the term is still very abstract, and the diversity among the associated terms is high. EST count ratios on lower annotation levels shift toward *C. clypeatus* mostly in terms of enzymatic activity on proteins and transcription machinery. The likely reason is the genetic heterogeneity of the *C. clypeatus* RNA sample. While all the *P. bernhardus* individuals originated from a comparatively small area around the island of Helgoland (Germany), the supplier for *C. clypeatus* could not provide us with more precise information about the region the animals were caught, but confirmed the possibility of different origins for the animals.

Beyond the abstract level of GO terms we focused on olfaction, the main sensory task of the antennules. As all previously described chemosensory genes (Grosse-Wilde et al., 2011) were present in the new assembly of our reference *M. sexta* dataset we assume the parameters set for assembly are reliable. Furthermore, it was possible to identify even lowly expressed genes such as IRs from the *M. sexta* data, which is based on substantially lower number of reads and total data. This indicates that the probability of missing entire gene families in the hermit crab datasets is negligible. The absence of receptors belonging to the OR family in the hermit crabs is consistent with findings in *Daphnia pulex* and the spiny lobster (Penalva-Arana et al., 2009; Corey et al., 2013) and supports the assumption that the OR superfamily developed only in the insect lineage of arthropods (Sanchez-Gracia et al., 2009). GRs, however, are present in the *Daphnia* genome but were not found to be antennaly expressed in the spiny lobster (Penalva-Arana et al., 2009; Corey et al., 2013). If the crustacean sense of taste is exclusively located on the walking legs as indicated in lobsters (Johnson et al., 1988), this could explain the absence of GRs from the antennal transcriptomes. The identification of the putative coreceptors IR25a and IR93a confirms the presence of the ancient IR nose in hermit crabs. Both IRs are conserved within the tetraconata, while the function and ligands of IR93a are unknown (Silbering et al., 2011). Nevertheless, its conservation indicates an importance across lineages, which seems to be independent from terrestrial or aquatic lifestyle. Most other candidate IRs cluster together with IRs from *P. argus* as decapod IR subgroups with no clear insect or *Daphnia* homolog. As little is known on the function of divergent IRs, it needs to be noted that their presence in hermit crab antennal transcriptomes confirms studies of the lobster antennae (Corey et al., 2013) indicating the existence of a new IR subgroup, as these IRs do not belong to the insect antennal IRs while divergent IRs are not expressed in the antennae of any insect studied so far. As clusters contain IR candidates from both hermit crabs and the aquatic *P. argus* it is rather likely that they have a function that is not depending on lifestyle. Contradictory to the expectation of specific olfactory-relevant receptors emerging after the transition from water to land, both hermit crabs seem to have a similar set of IRs expressed in the first antenna pair. Although no major screening of olfactory sensitivity including a large set of chemically different odors has been performed in aquatic hermit crabs, a recent electroantennographic study by Krång et al. showed that *C. clypeatus* detects almost exclusively water soluble odorants (Krång et al., 2012). These chemicals are putative cues available in the environment of both species. While genomic evidence of OBPs is present for most insect genera from the pea aphid (Zhou et al., 2010) to the silkmoth (Gong et al., 2009) but restricted to hexapods, CSPs were found in the *Daphnia* genome and could thus be expected in the hermit crabs as well (Vieira and Rozas, 2011). However, no CSPs are expressed in the antennal transcriptomes of either hermit crab. Binding proteins have been shown to be not restricted to olfactory tissues in insects and are likely involved in other functions as well (Pelosi et al., 2006). The absence of known families of binding proteins from the antennal transcriptomes indicates an independence of chemosensory detection from carrier proteins. This also fits the assumption that binding proteins are needed to transfer lipophilic odorants through the sensillum lymph, a function that might be dispensable for water soluble odorants (Kaissling, 2001; Leal et al., 2005). The EAG responses of *C. clypeatus* indicate a partial overlap with the odor spectrum evoking responses in IR expressing OSNs of coeloconic sensilla of *Drosophila* (Silbering et al., 2011; Krång et al., 2012). This raises the question if hermit crabs use the IRs related to the divergent IRs in insects, or altogether different receptors for the detection of the same chemicals. Since the divergent IRs are prime candidates, studies on expression and functionality of these receptors are needed to clarify whether or not they are involved in the detection of specific odorants. Following the described olfactory pathway in lobster we confirm details of the olfactory transduction pathway, indicating histamine-dependent regulation and a putative involvement of G-protein mediated signaling. Consequently, we propose a hermit crab olfactory transduction mechanism comparable to the one found in lobster (Hatt and Ache, 1994).

While the numerous cilia sensitive to mechanical stimuli on the crustacean antennules have been studied (Keil, 2012), molecular information is lacking. A recent study in *Drosophila* larvae screened for ESTs which are necessary to maintain the ability to sense touch events (Tsubouchi et al., 2012). Transcripts of receptors with similar features are also present in the hermit crab transcriptomes, including nompC and a ClCb channel in *C. clypeatus* and NMDA receptors in both datasets. The sense of touch might thus be mediated by related receptors in hermit crabs as in *Drosophila*.

The immune system of crustaceans is a subject of intense research, while the role of pathogen defense in the antennules has not been studied. We identified ESTs representing both more unspecific as well as pathogen-specific innate immune mechanisms. The presence of crustins, carcinins, C-type lectins, and anti-lipopolysaccharide factors (ALFs) in our datasets confirm the presence of pattern recognition mechanisms as well as production of AMPs in hermit crabs. Furthermore, *P. bernhardus* exhibits a higher diversity of AMPs than *C. clypeatus*, presumably due to either a larger number of potential pathogens, or a higher rate of exposure to small organisms recognized as non-self (i.e., algae) in the aquatic environment. The different types of cathepsins present might play a role in immune response, as cathepsin expression is upregulated upon viral challenge in shrimp (Robalino et al., 2007). The RNAi pathway, another defense mechanism against viral pathogens, is represented by homologs of the *Drosophila* RNA helicase Armitage and the conserved Tudor staphylococcal nuclease. Our findings thus demonstrate expression of various immune relevant genes in the antennae, with most of the known crustacean defense mechanisms present. This indicates the antennulae as a site of production and not only action of pathogen defense. A potential reason for this is the structure of the antennular cuticle. To allow the passage of odor molecules, the aesthetasc cuticle is comparatively thin at the exposed side (Stensmyr et al., 2005) and can thus easily be penetrated by invading microorganisms. Additionally the aesthetascs are immersed in a moist layer of a mucus-like substance (unpublished data). This could putatively promote the growth of microbes and therefore affect olfaction by production of odorants or clogging the entry sites of odorants with microorganisms. The importance of antimicrobial defense is also indicated by the raw sequencing data containing a high number of reads originating from bacteria, fungi, and algae.

Both hermit crab species are well-adapted to their habitats. The substantial changes in lifestyle by becoming terrestrial led to various morphological changes in the periphery of antennules (Ghiradella et al., 1968b) and an enlargement and reorganization of olfactory brain centers in *C. clypeatus* (Harzsch and Hansson, 2008). The changes regarding the OL of terrestrial hermit crabs are considered to be an adaptation to the aerial sense of smell and as an indication of fundamental changes in olfaction in general (Rittschof and Sutherland, 1986; Stensmyr et al., 2005). However, the molecular differences between the antennal transcriptomes of the marine *Pagurus bernhardus* and the terrestrial *Coenobita clypeatus* are overall small, indicating that the exhibited changes in function and morphology are mainly founded on changes in small numbers of genes. With respect to the main antennular function of chemosensing they display a similar set of IR candidates in count and sequence similarity. The number and full length of the receptor candidates, their ligand specificity, their expression pattern in the OSN populations and possible combinatorial effects need to be investigated further. No other chemosensory receptor candidates were identified in either of the species.

## Authors contributions

Katrin C. Groh and Ewald Grosse-Wilde carried out the bioinformatic experiments, data analysis and drafted the manuscript. Heiko Vogel participated in evaluation of data analysis. Heiko Vogel, Ewald Grosse-Wilde, Marcus C. Stensmyr, and Bill S. Hansson participated in study design, coordination and drafting of the manuscript. Katrin C. Groh wrote the paper. Reagents and analytic tools were provided by Bill S. Hansson. All authors read and approve the final manuscript.

## Conflict of interest statement

The authors declare that the research was conducted in the absence of any commercial or financial relationships that could be construed as a potential conflict of interest.

## Supplementary material

(a) Sequencing data (EBI study acc. nr. ERP002374, http://www.ebi.ac.uk/ena/data/view/ERP002374).

(b) ESTs have been deposited on ENA, accession numbers *P. bernhardus*: HABX01000001 to HABX01000085, *C. clypeatus*: HABY01000001 to HABY01000089.
